# Case report: A comatose patient with pregabalin overdose successfully treated with continuous renal replacement therapy

**DOI:** 10.3389/fmed.2023.1125653

**Published:** 2023-04-24

**Authors:** Visti Torbjørn Nielsen, Nelun Wijayasinghe, Lotte Christine Groth Høgberg, Søren Bøgevig

**Affiliations:** ^1^Department of Anaesthesia and Intensive Care, Copenhagen University Hospital - Bispebjerg and Frederiksberg, Copenhagen, Denmark; ^2^The Danish Poisons Information Centre, Bispebjerg Hospital, Copenhagen, Denmark

**Keywords:** pregabalin overdose, toxicology, continuous renal replacement therapy, intensive care medicine, coma

## Abstract

Pregabalin (PB) overdose causes mild symptoms and coma is rarely seen unless the patient has also ingested sedatives and/or has preexisting renal disease. We present a case report of a suicide attempt with PB where the patient presented in a comatose state that was successfully treated with continuous renal replacement therapy (CRRT). Treatment of PB overdose is usually supportive.

However, previous reports of PB overdose have been treated with intermittent hemodialysis (IHD) in patients with preexisting renal disease. The problem with IHD is that it is only available in specialist centers and unsuitable for unstable patients. In the following case report, the patient presented to the emergency department (ED) unconscious and hypotensive. It was thought that the patient tried to commit suicide by taking an overdose of zopiclone tablets, as empty packets of zopiclone tablets were found beside the patient. There was no effect with flumazenil treatment, so the patient was intubated, mechanically ventilated, and admitted to the intensive care unit (ICU) where inotropic support was started. Despite supportive therapy, there was no improvement in the patient’s condition. Further investigation into the patient’s medical records uncovered prescriptions of PB. Based on this finding, plasma PB levels were measured and found to be 20 times the upper limit of the therapeutic reference range. CRRT was instituted and after 6 h of treatment the patient woke up. Hospitals with ICUs often have CRRT available in their units whereas IHD is less readily available. This case report demonstrates that CRRT is an effective method for treating PB overdose in an unconscious unstable patient that was unsuitable for transfer to another hospital.

## Introduction

Pregabalin (PB) is a synthetic, structural derivative of the inhibitory neurotransmitter γ- aminobutyric acid (GABA), however is inactive at GABAA and GABAB receptors. PB binds to the α2δ-subunit of voltage-dependent calcium channels in nerve terminals modulating calcium influx leading to a reduction in the release of several neurotransmitters such as glutamate, noradrenaline, serotonin, dopamine and substance P (1). PB is prescribed for partial seizures in patients with epilepsy, central and peripheral neuropathic pain, and more recently generalized anxiety disorder (1, 2). The expanded use of PB has led to an increased rate of prescriptions over the last decade and thus an increased likelihood of suicide attempts and overdoses with PB (2).

Pregabalin-only overdose generally causes mild symptoms, although seizures and coma have been rarely reported (3). The incidence of severe pregabalin poisoning with coma and signs of encephalopathy is limited to a few case reports, however patients had co-existing renal failure and/or had ingested sedatives (4, 5). Intermittent hemodialysis (IHD) has been successfully used in the management of pregabalin toxicity in a patient with preexisting renal disease and in a child with no underlying comorbidity (6, 7).

This report presents a case of severe poisoning with pregabalin successfully treated with continuous renal replacement therapy (CRRT) combined with supportive intensive care. Plasma pregabalin concentration measurements were used to monitor the response to CRRT.

## Case description

A 66-year-old female was brought to the Emergency Department (ED) unconscious and hypotensive. Her medical history included schizophrenia and chronic obstructive pulmonary disease, but no renal or liver disease.

The patient was found unconscious by her community nurse, next to empty packets of zopiclone tablets. Initial assessment indicated attempted suicide with the ingestion of up to 30 tablets zopiclone 7.5 mg (total dose 225 mg). The community nurse had seen the patient 18 h prior to admission to the ED and stated that the patient had been her normal self. On admission to the ED, an arterial blood gas showed a mixed respiratory and metabolic acidosis: pH 7.16, pCO_2_ 9.1, pO_2_ 16.1, BE – 4.4, HCO-_3_–9.6, blood glucose 15 mmol/L, lactate 3.6 mmol/L. The patient was also hyperkalemic with a potassium level of 6.9 mmol/L (reference 3.5–4.4 mmol/L). This was treated with an intravenous i.v Glucose-Insulin infusion and i.v calcium gluconate and the potassium level fell to 6.2 mmol/L in the ED. The blood pressure was 110/47 mmHg, heart rate 107 beats/min increasing to 150 beats/min and the patient’s temperature was 37.8°C. The ECG showed no evidence of arrhythmias or ischemia.

**Figure 1:**
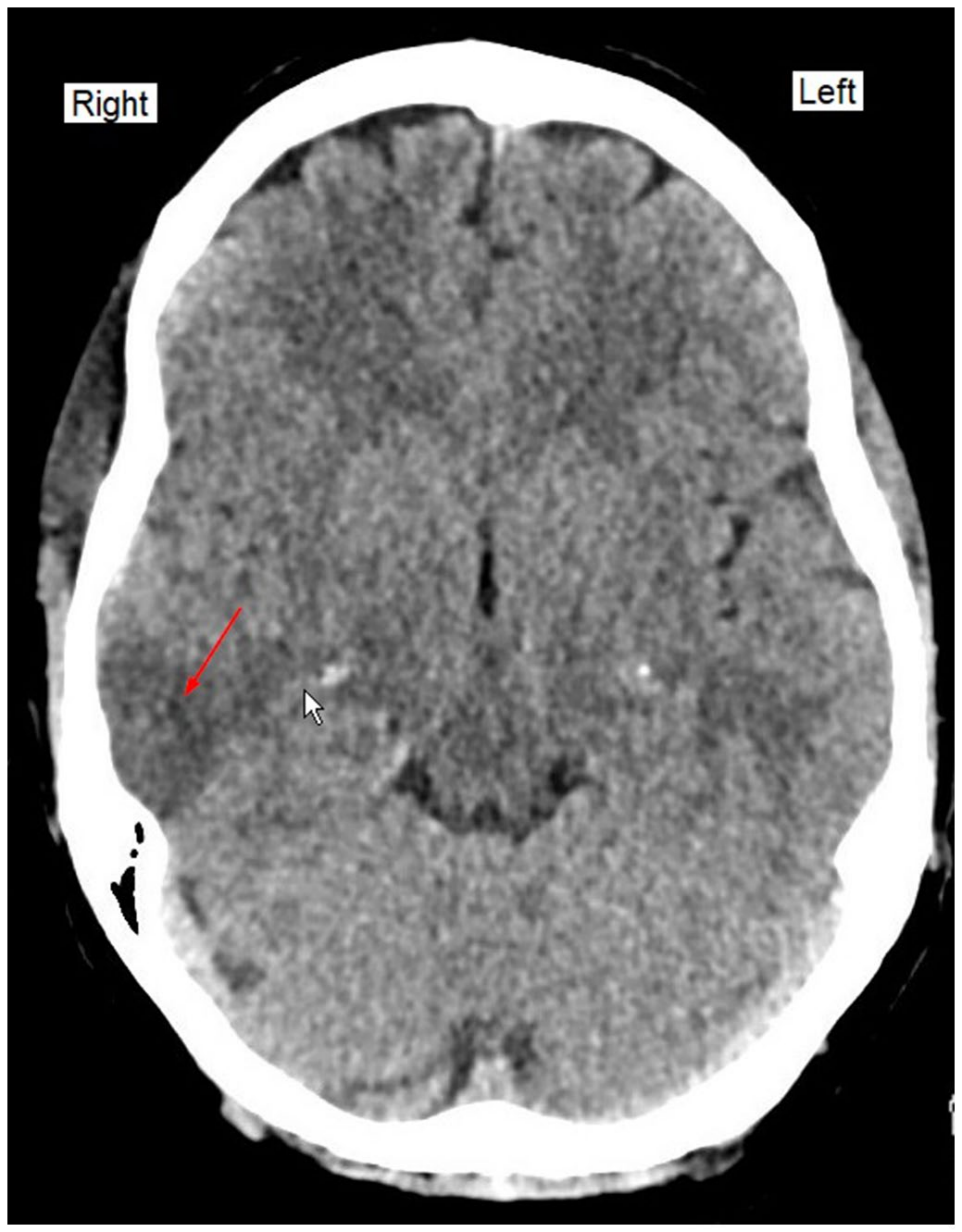
CT (Computed Tomography) of the brain on admission. Red arrow indicate the possible minor acute or subacute infarcts in the right temporal lobe.

Administration of 0.2 mg flumazenil i.v had no effect, therefore the patient was intubated, mechanically ventilated and admitted to the intensive care unit (ICU). A CT of the brain, chest, and abdomen indicated possible minor acute and subacute infarcts in the right temporal lobe, right sided pneumonia and hepatic steatosis (Figure 1).

The patient received 50 grams of activated charcoal *via* nasogastric tube and was sedated with propofol and remifentanil. Norepinephrine was initiated in the ICU with a maximum of 0.37 microgram/kg/min within the first 60 minutes but was reduced over the next few hours due to a mean arterial blood pressure of 65 mmHg being established.

Blood samples (normal ranges in parentheses) following admission revealed signs of rhabdomyolysis with renal injury, eGFR/1.73 M^2^ 20 mL/min (normal >60 mL/min), Creatinine 217 mmol/L (50–90 mmol/L), urea 7.2 mmol/L (3.1–7.9 mmol/L), myoglobin 30,000 μg/L (19–49 μg/L) decreasing to 19,800 μg/L and 11,700 μg/L on day 2 and day 3 respectively, creatinine kinase (CK) 10,800 U/L (105–205 U/L), increasing to 34,800 on day 2, lactate dehydrogenase >1800 U/L (105–205 U/L), bilirubin 4 μmol/L (5–23 μmol/L), CRP 5 mg/L (< 15 mg/L), Haemoglobin 9.5 mmol/L (7.3–9.5 mmol/L), leucocytes 14×10^9^/L (3.5–8.8 E9/L), alanine transferase 677 U/L (10–45 U/L), Neuron-Specific Enolase 27 μg/L (< 16.3 μg/L). Ethanol, salicylate and paracetamol were all below the detection limit.

Both transoesophageal echocardiography (TEE) and transthoracic echocardiography (TTE) were near to normal with a left ventricular ejection fraction of 60%.

A neurologist examined the patient 6 h after admission to the ICU and assessed a FOUR (Full Outline of UnResponsiveness) score of 2 (E0, M0, B2, R0), the sedation with propofol had just stopped 45 min prior to the examination and continued sedative effects of propofol were suspected. The sedative medication was stopped, but the patient remained unconscious during the following 24 h.

Neurological reexamination on day 2 showed a FOUR score of 3 (E0, M0, B2, R1). CT-angiography was performed which showed no evidence of arterial thrombi but demonstrated possible minor infarcts in the right temporal lobe and frontal parietal region. Electroencephalogram (EEG) readings were also taken which showed no signs of non-convulsive status epilepticus. Lumbar puncture was performed with clear cerebrospinal fluid and no signs of infection or hemorrhage. Thus, a drug overdose was suspected prior to the right side temporal and frontal parietal subacute infarctions, corroborated by the clinical signs of deep areflexia, universal hypotonia, no ocular-cephalic-reflexes, but with pupils equal in size and reactive to light.

Further investigation into the patient’s medical notes revealed prescriptions for risperidone, inhalers (bronchodilators and steroids) and PB. Therefore, PB overdose was now suspected, and measurement of plasma levels confirmed this.

**Figure 2:**
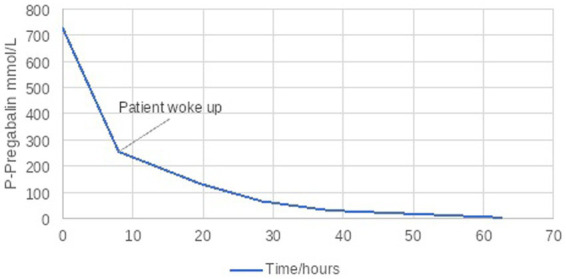
P-Pregabalin during CRRT.

Plasma concentration of PB was measured 36 h post admission and was found to be 727 μmol/L, more than 20-fold the upper therapeutic reference range limit (range 10–35 μmol/L). Therefore, CRRT was initiated to eliminate PB. The patient’s bodyweight was 74 kg, height 169 cm and BMI 25.9 kg/m^2^. CRRT was therefore initiated with a “Fresenius Multifiltrate Pro dialyzer” with an Ultraflux AV1000S filter, set to CVVHD (Continuous veno-venous hemodialysis) mode using a double lumen Fresenius ProVen^®^, 11.5 French catheter through right internal jugular vein. The blood flow rate was set at 100 ml/min and dialysate flow at 2000 mL/h. Regional citrate anticoagulation was used as anticoagulation of the extracorporeal circuit. After 6 h, the patient suddenly regained consciousness, and 8 h after CRRT was initiated plasma-pregabalin concentration was 255 μmol/L. CRRT was continued and plasma-pregabalin concentration decreased to 133 μmol//L and 66 μmol/ L after 19 and 28 h, respectively (Figure 2).

Twenty-four hours after CRRT was initiated the patient had a GCS of 14, stable circulation and was extubated. The CVVHD continued for two more days, during which the rhabdomyolysis resolved and the kidneys regained full function.

The last neurological examination before discharge from the ICU was performed 3 days after the patient was admitted to the ICU and here the patient had a GCS **13–14** (E3, V5, M5-6) and a FOUR score of **15–16** (E3-4, M4, B4, R4) The patient was discharged from the ICU on day 6 to the neurology ward for rehabilitation. It was difficult to decipher whether the patient had a cognitive deficit from the overdose. After a month on the neurology ward, the patient was transferred to the psychiatric unit.

## Discussion

Continuous renal replacement therapy (CRRT) and intermittent hemodialysis (IHD) are forms of extracorporeal renal-replacement therapy (ECRT). In most countries, CRRT is only used in ICUs. CRRT dialyzes the patient slower than conventional IHD. CRRT is used for treatment of acute or chronic renal failure, hypervolemia and the elimination of toxic substances. Reduced renal function has been suggested to be the underlying cause of pregabalin (PB) overdose/ toxicity in case reports and the current recommended extracorporeal treatment for PB toxicity is IHD (5). IHD, is not readily available in all hospitals because it requires expertise within nephrology and technical support, whereas CRRT is accessible in hospitals with ICUs. Furthermore, interhospital transfer of the critically ill patient, such as the severely poisoned patient, can be a high-risk procedure (8).

The pharmacokinetics of PB enables ECRT for its elimination, as pregabalin does not bind to plasma proteins, has a small molecular weight of 159.23 g/mol and a small volume of distribution (0.4–0.6 L/kg). Pregabalin is readily absorbed and has an oral bioavailability of 90%. It is not significantly metabolized, with 98% excreted unchanged in the urine and a linear pharmacokinetic profile with an elimination half-life of approximately 6 h (4,9). Pregabalin elimination is nearly proportional to creatinine clearance and PB renal clearance is estimated to be 67.0 to 80.9 mL/min in young healthy subjects (9). Our patient had an elimination half-time of 6.7 to 10.2 h and a clearance between 45 and 70 mL/min. (Table 1) once CRRT was started. Thus, PB elimination could only be achieved once kidney function was regained.

**Table 1:** Calculation and elimination half-time and clearance of PB.

Time	Plasma PB	t½ (hours)	CL (mL/min)
0	727		
8	255	6,7	70
19,6	133	10,2	45
28,4	66	8,7	53
38	31	8,8	53
62,5	4	8,3	56

Evaluation of the dose-toxicity relationship for PB is described by Rietjens et al. and there seems to be a relation between consciousness and degree of poisoning. Though it has been reported, a GCS of 6 or less at hospital admission due to pregabalin overdose in isolation is seldomly observed (3). In addition, management with intubation and/or inotropes is rare with sole pregabalin intoxication. Pregabalin overdose as a single-substance overdose is usually benign, although seizures may occur, but when sedatives are co-ingested, coma is more commonly observed (2).

A strength of this case is that as soon as PB overdose was suspected, plasma levels could be measured and treatment instigated. Furthermore, plasma levels of PB were used to guide treatment and thus adds to our knowledge of the pharmocodynamic effects of PB. A limitation of this case is that it is unclear whether this was a mixed overdose of PB and zopiclone. If the 30 tablets of zopiclone that were mentioned above were ingested, then this amount was estimated to be low to moderate therefore unlikely to have induced a coma, but measurements of plasma levels of zopiclone was not performed to confirm this. Another limitation is the dose of flumazenil that was used to diagnose a zopiclone overdose. Flumazenil can be used successfully for zopiclone overdoses but in our case, there was no effect. This could be due to the flumazenil dose being too small and thus inadequate in an overdose. However, when the patient first presented to the ED, there was a suspicion that the patient had had a seizure therefore flumazenil was used cautiously. Another contributing factor to the patient’s comatose state could have been the rhabdomyolysis, although it would be unlikely to have been the sole reason for the cerebral dysfunction.

A limitation in the treatment of the PB overdose could also be that the dialysate flow was too low. In this case the dialysate flow started at 2000 mL/h. With our current knowledge an increased dialysate flow from the start may have been better because dialysate flow is a limiting factor for clearance of small molecules that do not bind to plasma proteins and have small volumes of distribution. With rapid decreasing plasma-PB concentrations after CRRT initiation, we believe CRRT is a sufficient alternative to IHD in the acute severe PB overdose setting. In an unconscious patient with acute renal failure, CRRT may shorten a patient’s stay in the ICU if it is initiated early. This does mean that treatment with IHD is delayed but the patient can also be stabilized and thus transferred to a hospital with expertise in IHD when appropriate.

Our conclusion is that the use of CRRT in this case led to reversal of neurotoxicity and regain of consciousness due to the accelerated elimination of pregabalin.

## Data availability statement

The data analyzed in this study is subject to the following licenses/restrictions: the data used in this case report is availiable in the patients medical record at the hospital. But all the data of interest for this case is reproduced in the casestory. Requests to access these datasets should be directed to visti.torbjoern.nielsen@regionh.dk.

## Ethics statement

Written consent was obtained from the individual(s) for the publication of any potentially identifiable images or data included in this article.

## Author contributions

VN: staff specialist in anesthesiology at the intensive care unit and mainwriter and the doctor who was treating the patient the first days, collecting all data and setting the team. NW: staff specialist in anesthesiology at the department and helping with the manuscript. LH: pharmacist at the The Danish Poisons Information Centre Bispebjerg Hospital Denmark - Insight in severe overdoses and toxicology and helping with the manuscript. SB: senior consultant in anesthesiology at the The Danish Poisons Information Centre Bispebjerg Hospital Denmark - Calculations, insight in CRRT and helping with the manuscript. All authors contributed to the article and approved the submitted version.

## Conflict of interest

The authors declare that the research was conducted in the absence of any commercial or financial relationships that could be construed as a potential conflict of interest.

## Publisher’s note

All claims expressed in this article are solely those of the authors and do not necessarily represent those of their affiliated organizations, or those of the publisher, the editors and the reviewers. Any product that may be evaluated in this article, or claim that may be made by its manufacturer, is not guaranteed or endorsed by the publisher.
